# Validation of PARADISE 24 and Development of PARADISE-EDEN 36 in Patients with Dementia

**DOI:** 10.3390/ijerph19116949

**Published:** 2022-06-06

**Authors:** Francesco Talarico, Carolina Fellinghauer, Giuseppe Andrea De Biase, Pietro Gareri, Sebastiano Capurso, Paolo Moneti, Angela Caruso, Valentina Chiatante, Emanuela Gentile, Monica Malerba, Laura Marsico, Maria Mauro, Maria Magro, Andrea Melendugno, Fabio Pirrotta, Luana Putrino, Carla Putrino, Anna Propati, Vincenzo Rotondaro, Fausto Spadea, Angela Villella, Alba Malara

**Affiliations:** 1 Fondazione ANASTE-Humanitas via dei Gracchi, 00192 Roma, Italy; france.talarico12@gmail.com (F.T.); giuseppeandreadebiase@gmail.com (G.A.D.B.); 2 Data Fittery GmbH, 4665 Oftringen, Switzerland; carolina.ballert@gmail.com; 3 Center for Cognitive Disorders and Dementia (CDCD), Catanzaro Lido, ASP Catanzaro, 88100 Catanzaro, Italy; pietro.gareri@me.com; 4 RSA Bellosguardo, ANASTE Lazio, 00053 Civitavecchia, Italy; seb.capurso@gmail.com; 5 RSA Villa Gisella, ANASTE Toscana, 50100 Firenze, Italy; p.moneti@gruppozaffiro.it; 6 RSA La Quiete, ANASTE Calabria, 87040 Castiglione Cosentino, Italy; angelacaruso655@gmail.com (A.C.); marsicolaura@libero.it (L.M.); 7 Casa Protetta Villa Azzurra, ANASTE Calabria, 87070 Roseto Capo Spulico, Italy; v.chiatante@gmail.com (V.C.); annapropy@tiscali.it (A.P.); tdrenzo@81gmail.com (V.R.); 8 Casa Protetta Madonna del Rosario, ANASTE Calabria, 88046 Lamezia Terme, Italy; em.gentile@libero.it (E.G.); angela03villella@alice.it (A.V.); 9 Centro Residenziale e di Riabilitazione San Domenico, ANASTE Calabria, 88046 Lamezia Terme, Italy; monicamalerba@alice.it (M.M.); melolulu@virgilio.it (L.P.); carlaputrino@tiscali.it (C.P.); 10 RSA Casa Amica, ANASTE Calabria, 88050 Fossato Serralta, Italy; mauromaria565@gmail.com (M.M.); mariamagro66@gmail.com (M.M.); spadeafausto@libero.it (F.S.); 11 O.P.S.A. Opera Provvidenza Sant’Antonio, C.S. Casa Madre Teresa di Calcutta, 35030 Sarmeola, Italy; a.melendugno@operadellaprovvidenza.it; 12 Fondazione Betania Onlus, 88100 Catanzaro, Italy; fapirro@gmail.com

**Keywords:** PARADISE-EDEN scale, ICF, dementia, functional state, long term care

## Abstract

Dementia was one of the conditions focused on in an EU (European Union) project called “PARADISE” (Psychosocial fActors Relevant to brAin DISorders in Europe) that later produced a measure called PARADISE 24, developed within the biopsychosocial model proposed in the International Classification of Functioning Disability and Health (ICF). The aims of this study are to validate PARADISE 24 on a wider sample of patients with mild to moderate dementia to expand PARADISE 24 by defining a more specific scale for dementia, by adding 18 questions specifically selected for dementia, which eventually should be reduced to 12. We enrolled 123 persons with dementia, recruited between July 2017 and July 2019 in home care and long-term care facilities, in Italy, and 80 participants were recruited in Warsaw between January and July 2012 as part of a previous cross-sectional study. The interviews with the patient and/or family were conducted by health professionals alone or as a team by using the Paradise data collection protocol. The psychometric analysis with the Rasch analysis has shown that PARADISE 24 and the selection of 18 additional condition-specific items can be expected to have good measurement properties to assess the functional state in persons with dementia.

## 1. Introduction

Dementia is a syndrome that causes deterioration of cognitive functions that also affects the quality of life, and causes distress in families and communities, increasing demand for healthcare and social services. Nowadays it is a global epidemic involving 50 million patients worldwide representing the 7th leading cause of death [1].

In May 2017, the World Health Assembly endorsed the “Global action plan on the public health response to dementia 2017–2025” [2], recognizing dementia as a public health main target. Among the tools developed by WHO, the International Classification of Functioning, Disability and Health (ICF), systematically groups different health domains and health-related domains (e.g., what a person can do or does do when he or she has a given health condition). Indeed, clinical information alone is not able to depict the health experience of a person with disabilities, thus limiting the possibility to plan adequate health services, required level of assistance, use of appropriate aids and social integration, etc. [3].

As for dementia, clinicians should be able to assess functional performance, where this information is integral to understanding health and for the optimal provision of clinical care and implementation of individual measures of rehabilitation designed to improve executive function. Functional status can be conceptualized as the ability to perform self-care, self-maintenance, and physical activity. A person with dementia usually requires help with more complex tasks, such as managing bills and finances or simply maintaining a household. Good functional performance is fundamental for elderly people to maintain independence and avoid institutionalization [4].

In dementia patients, functional capacity and functional impairment are not a uniform construct nowadays; rather, they are multifaceted and can be measured with various clinical instruments. Many scales have been validated for use in patients with Alzheimer’s disease (AD) in order to characterize functional impairment and to evaluate the clinical staging of dementia or the efficacy of treatment. Several methods, such as psychometric testing, evaluate only one dimension, typically cognition, mood, communication capability, or behavioral problems [5,6,7]. Therefore, many combinations of simpler psychometric and behavioral evaluations have been variously used for the assessment of dementia patients with different degrees of severity. Reliability, validity, and correlational data are discussed if the test is used alone or in a different combination of assessing methods [8].

The ICF represents a good chance for a better understanding of the functional issues associated with dementia. ICF is a classification that can provide a basis to select relevant categories from functioning domains that describe functioning with dementia. To date, an ICF core-set containing the full spectrum of the problems of patients with dementia does not exist. An assessment tool, ideally personalized, multidimensional, and a complete scale for easier and more suitable use in dementia patients is also still missing.

The assessment of disability in patients with dementia, through the ICF model, allows for the ability to formulate a dynamic functional profile and identify the associations among the health condition, comorbidities, environmental and personal factors with disability levels. A previous study was carried out across a network of Calabrian Long Term Care (LTC) Facilities conducted by ANASTE (Associazione Nazionale Strutture e Territorio), which aimed to describe disability by means of ICF profiles for the Activity and Participations domains in a cohort of residents suffering from moderate-severe Alzheimer’s Disease (AD) and Vascular Dementia (VD), showed that the goals of care processed according to the indicators of ICF Capacity and Performance, allow an improvement of disability assessment in the two forms of dementia. The use of the ICF can guide the planning of health care interventions for patients with dementia, taking into account important aspects of daily life, usually less considered, such as communication, social relationships, and leisure activities [9].

Dementia was listed as one of the conditions focused on in an EU (European Union) project called “PARADISE” (Psychosocial fActors Relevant to brAin DISorders in Europe) that later produced a measure called PARADISE 24. This scale was used to assess the impact of psychosocial difficulties on the lives of people with nine different brain disorders (dementia was one of these conditions) through 24 questions [10,11]. The severity of these Psychosocial Difficulties (PSDs) depends on many factors including social, physical, and political environments within the biopsychosocial model proposed in the ICF [12]. The items included in PARADISE 24 presented generally good measurement properties and a few limitations [11]. The authors further advised testing the instrument on a larger number of people affected by specific brain disorders to complete the process of validation on larger sample sizes. Furthermore, instead of one metric for brain disorders, it could be necessary to develop scales dedicated to specific neurologic or psychiatric conditions [11].

The ANASTE Humanitas Foundation, a scientific branch of ANASTE, started a project aimed, first of all, at testing PARADISE 24 on a wider sample of patients with mild to moderate dementia in different care settings (facilities, day-care centers, and homes). Further, the project expanded PARADISE 24 by defining a more specific scale for dementia and adding to the 24 questions of PARADISE, 12 questions specifically selected for dementia.

The aims of this study are to validate PARADISE 24 on a wider sample of patients with mild to moderate dementia, to corroborate the 18 condition-specific selected items, and to gather recommendations to reduce the set of items towards a PARADISE-EDEN (Empowering DEmentia Narrative), 24 + 12 items version, specific for dementia patients. The specific aims are to test the metric properties of the 24-items and the 18-items scale through a psychometric analysis with the Rasch analysis [13].

## 2. Methods

### 2.1. Study Design and Samples

This study is aimed at testing the metric properties of the PARADISE 24 items, selected for the assessment of brain injuries, in a population of persons with Dementia. In addition to these 24 items, the EDEN collection included 18 functioning items from the original 64 items version that were expected to be more sensitive in a dementia population (Table 1: Item 25 to Item 42).

In order to reach the second target, it is important to take into account that the original questions addressing 64 PSDs were finally reduced in previous studies to include only 24 items in the PARADISE 24 metric [11]. In fact, the PARADISE 24 assessment scale kept the items with the best psychometric properties and most representative for the assessment of functioning across the nine brain disorders. It can so be expected, that the original pool still contains items suitable for the assessment of a single condition such as dementia. In that sense, further items useful to develop a scale specific for dementia were chosen from the 40 discarded items that were originally included in the PARADISE research project. Eighteen additional items were selected based on the expertise of clinical and health care professionals, with wide experience in treating persons with dementia in a focus group exercise. The additional items were selected from the original 64 items pool used in PARADISE. Items from the pool were selected if they represented a problem for at least 33% of the patients with dementia. These items were further narrowed considering only those ranked in the first six positions among the most common items reporting difficulties in patients with dementia. Thus, a total of 18 items were selected that should ideally be reduced to 12 items to be more manageable, resulting in a set of 24 + 12 items, called PARADISE-EDEN (Empowering DEmentia Narrative), suitable for assessment of patients with dementia.

The data used for this study came from two different data collections. Originally, only data collected in a multi-setting cross-sectional study carried out in Italy between July 2017 and July 2019 in the realm of the PARADISE-EDEN project was intended for this study. This collection included data from N = 123 persons with dementia that were recruited between July 2017 and July 2019. The persons had a clinical diagnosis of dementia or a more specific diagnosis of probable or possible VD or AD [14,15,16]. The clinical diagnosis of dementia was firstly investigated through a detailed personal interview, as well as family history and, subsequently, confirmed by the administration of psychometric tests. The cognitive evaluation was conducted by Folstein’s Mini-Mental State Examination (MMSE) [17]. According to MMSE, the patients were affected by slight cognitive impairment, if MMSE scores ranged from 21–23. The functional state was evaluated by use of the Activity Daily Living scale (ADL) [18], according to which a lower score indicates a worse functional state. The EDEN data collection was conducted in conformity with the ethical principles of the European Commission Research Ethics Committee. The study and the informed consent used were approved by the Ethics Committee of Calabria Region.

A sample size of N = 123 appeared small to perform a reliable Rasch analysis. To increase the power of the psychometric analysis, it was decided to add the observations from the PARADISE survey that included adult participants having a main ICD-10 diagnosis of dementia. The PARADISE survey aimed to understand the impact of brain disorders on people’s lives, based on the Psychosocial Difficulties (PSDs) that are experienced in common across brain disorders. The data were collected as part of a cross-sectional study using convenience sampling and retained N = 80 participants who were at the Institute of Psychiatry and Neurology in Warsaw between January and July 2012. The interviews with the patient and/or family were conducted by health professionals alone or as a team, specifically trained on ICF, by using the PARADISE data collection protocol. More information about the PARADISE data can be found in other papers [10,11]. The PARADISE data collection was conducted in conformity with the ethical principles of the EC Research Ethics as well as by the Ethics Committee of the Institute of Psychiatry and Neurology in Warsaw, Poland. Participants were informed of the purpose and rationale of the study and signed a consent form.

Differences in the functioning of the questionnaire items based on the data collection were undertaken as part of a so-called analysis of differential item functioning (DIF), as further explained in the next section.

### 2.2. Statistical Analysis

Psychometric analyses using the Rasch analysis, here more specifically a Partial Credit Model (PCM) for polytomous data [19], typically test a series of metric assumptions to determine the good fit of the psychometric model to the data. A Rasch analysis observes if the data collected through an assessment form with ordinally scaled items present specific characteristics essential for measurement. In practice, beyond the general fit to the model, the measurement assumptions that are tested are: reliability, targeting, item fit, monotonicity, absence of local item dependencies (LID), unidimensionality, and absence of differential item functioning (DIF) [20]. If these assumptions are met, a test form is considered “fit” for measurement and delivers an interval scaled raw score [21]. The psychometric analyses of the different selection of PSD items, i.e., PARADISE 24, EDEN 18, and the two sets combined, followed a strictly identical approach in terms of assumptions that were tested, the methods that were applied, therefore, and the interpretation of the statistical outputs.

*Reliability:* When reporting psychometric analyses, the reliability coefficient is considered a critical statistic to support the quality of an assessment scale [22]. The Cronbach α is the most widely used measure of reliability and is a measure of the internal consistency of the data, i.e., how well the items work to describe one construct [23]. In modern test theory, reliability is discussed in terms of separation, specifically the person and item separation. Two ways to formalize the separation are the Separation Indices and the Separation Coefficients. Typically, reliability is supported with an Index of 0.8, indicating good reliability and excellent reliability with values above 0.9 [20]. A low Item Separation Index means that the sample is not large enough to locate the items on the latent variable. A low Person Separation Index indicates that the measure fails to discriminate between different levels of ability. The Person and Item Coefficients describe the ‘true’ spread of items or individuals along the measurement continuum. A Person Separation Coefficient of 1.5 represents an acceptable level of separation and is considered the minimum required to divide the sample into two distinct strata (i.e., low and high levels of functioning). A Person Separation Coefficient of 2.0 represents a good level of separation. Strata can be derived from the Separation Coefficient and indicate the expected number of statistically distinguishable levels of functioning that the scale can assess [24].

*Targeting:* Good targeting of a test form indicates that the difficulty of an assessment matches the ability level of the respondents. In general, targeting requires that the mean item difficulty and mean person ability correspond, supporting that the test form is neither too difficult nor too easy for the respondents. The dispersion of the ability and difficulty estimates should also overlap, and ideally, the assessment spectrum should address the ability continuum of the sample.

*Item Fit* is supported when responses to test items are a function of the abilities of the respondents and the item difficulties. The good fit of the model function to the data is reflected through the analysis residuals. If the items are fitting the PCM, the mean squared residuals (MSQ) values per item are expected close to one, also called the Outfit. The Infit is a weighted total statistic for the MSQ that is less sensitive to outliers than the Outfit. In this analysis, the cut-off for good fit was determined using an approach that takes into account the sample size so that Outfit values > 1.42 and Infit values > 1.14 were indicative of misfit [25]. 

*Monotonicity* implies strictly increasing difficulty estimates for the item response thresholds. In practice, disordered response thresholds in some items are more the rule than the exception. Especially, items with many response options and poorly discriminating middle categories tend to show breaches of the threshold ordering [26,27]. The Paradise items were originally developed with five response options, but metric analyses showed that to respect the intended ordering, three options would perform better [10]. In that sense, the data collected in Italy used three response options, 1 = None, 2 = Mild to Moderate, and 3 = Severe to Extreme functioning problems. The additional original Paradise data, still rated on the 5-point response scale, was recoded accordingly. We also note here, that Item 16 assessing ’Problems in grooming, dressing, toileting or eating” was assessed as separate variables in the original data collection. They were now, aggregated into one item using the maximum score [11] and further recoded into three response options to be in line with the Eden collection.

*Local Item Dependencies:* LID often occurs when items are redundant and measure approximately the same or very similar aspects of a latent construct. Typically, high positive correlations of the standardized Rasch residuals, also named the $Q_{3}$ fit statistic [28], indicate LID. Negative residual correlation can reveal the multidimensionality of the form. LID leads to inflated reliability estimates and may give a false impression of reliability [29,30]. For this analysis, with a sample size of N = 201, a non-conservative cut-off of r=0.3 was used to mainly flag items with a higher dependency.

*Unidimensionality:* For a valid score, the test form should measure only one construct. With several separate dimensions, one total score is not valid anymore. Principal Component Analysis (PCA) of the standardized Rasch residuals tests the unidimensionality by searching for non-random patterns in the analysis residuals [31]. A first eigenvalue < 1.8 was deemed indicative of unidimensionality.

*Differential Item Functioning:* The analysis of Differential Item Functioning (DIF) flags variables that lack invariance of the item difficulty across subgroups [32]. A two-way ANOVA was used to test for uniform and non-uniform (DIF variable × score level) DIF including the variables: gender, age grouped (1 = (70,80], 2 = (80,85], 3 = (85,95] years), the data collection (PARADISE versus EDEN), the diagnosis (vascular dementia, Alzheimer’s or other type of dementia) and Residency (home or inpatient care). The DIF analysis with ANOVA was corrected for multiple testing using the Benjamini-Hochberg correction for the false discovery rate [33]. For the DIF analysis, missing values were inputted to avoid the deletion of cases with missing values and consequent reduction of the already smaller sample size. The data were imputed using the R-Package missForest [34] which provides a robust imputation algorithm for data of mixed types.

## 3. Results

*Sample and Item Descriptives:* Table 2 shows the descriptive statistics of the sample and stratified by data collection, i.e., PARADISE versus EDEN. Groupwise significance tests are performed to compare the data collections using the *t*-test for continuous variables and $\chi^{2}$-Test for categorical variables as a default [35]. The mean age was 82.55 years (SD = 6.95), and the mean ages in the two surveys were close to the sample average. The gender distributions and marital status also did not differ significantly across data collections. Most participants were female (76%), about half of the participants were widowed (56.7%), and more than a quarter were married (26.4%). In the Paradise data, most participants had completed high school or an equivalent level of education (40%), and about one-third (27.5%) had completed university. In the Eden collection, about one-third (27.3%) were illiterate, and most of the participants (55.4%) had completed primary school. Another significant difference was observed for the living situation where only 27% were living at home in the Eden collection, while for Paradise it was 87.5%. Overall, the entire sample presented a 44.3% diagnosis of Alzheimer’s disease and 47.4% vascular dementia, and the remaining participants had other forms of dementia. The percentages of diagnoses significantly differ across the two data collections with 61.3% of Alzheimer’s disease in the Paradise collection and 32.5% of the same diagnosis in the Eden collection. The mean MMSE was 21.1 (SD = 2.89) in the Paradise collection and 18.03 (SD = 3.83) for the Eden collection indicating on average mild to moderate levels of dementia. With regard to activities of daily living (ADL), most participants of the Paradise survey indicated being neither satisfied nor dissatisfied with their capacity to perform ADLs (41.2%). In the Eden data collection, most participants indicated a severe dependence in ADL (41.7%).

Appendix A presents the descriptive statistics, i.e., frequency and percentages, of the responses to the questionnaire items, also stratified by data collection. The numbers in the Appendix A show that the items were answered very differently and point to different levels of functioning between the two data collections. An explanation could be the significantly different percentages of persons living at home, where the Paradise sample included participants with higher levels of independence. This difference is not expected to harm the calibration of the questionnaire items as the mode of functioning of the items can still be apprehended independently of the levels of functioning. What matters is that the probability of an answer is a function of the difficulty of the question and the level of functioning of the respondent.

*Missing Values:* A few items showed a very high percentage of missing values. Item 17 ‘Difficulty with sexual activities’ had the highest number of missing values with a total N = 93 (46.3%) missing values. Among these N = 68 (85%) belonged to the Paradise collection and N = 25 (20.7%) to Eden. The lower percentage of missing values in the Eden data collection may be due to a higher emphasis on affection and desire to describe the libido than in the original data collection. Item 18 ‘Staying by yourself’ has also a noticeable number of missing values with N = 32 (15.9%) missing values overall, N = 18 (22.5%) missing values in the Paradise collection, and N = 14 (11.6%) in Eden. Item 22 ‘Difficulty in day-to-day work or school’ had a total of N = 77 (38.3%) missing values, with N = 54 (67.5%) in the original Paradise data collection, and N = 23 (19%) in the Eden collection. The Eden collection had a more relaxed definition of work than Paradise, where supervised occupations within the health care institution, for example, were considered as work.

*Reliability:* As shown in Table 3, the reliability of the PARADISE 24 items was very good, with an index of 0.9 and α of 0.91. The supplementary EDEN 18 items, when calibrated separately, showed lower but still good reliability with an index of 0.88 and α of 0.91. In the joint analyses with 42 items, the reliability index of 0.94 and α 0.95 also support a very high reliability. However, it must be noted that at this stage, without further adjustments for local dependencies, the reliability estimates are inflated. If addressing these, the Person Separation reliability will decrease. However, starting at such a high level of reliability, at least some acceptable reliability can be expected if aggregating dependent items. All separation indices lie above 2.5 at this stage and indicate that several levels of functioning are distinguished statistically. More specifically, the strata indicate that more than four levels of functioning were found in the separate calibrations and at least six levels when analyzed jointly. The Paradise 24 items differentiate a little below six levels of functioning (5.89) while the Eden 18 items 6.58, and all items together 6.08.

*Targeting:* All three approaches presented good targeting with a difference between the mean level of difficulty and mean level of ability below 0.5 logits. The highest discrepancy is found in the analysis with the EDEN 18 items and represents 0.23 logits. The higher difference may be due to the specific targeting of dementia that this selection represents, making it a scale where the respondents were more likely to indicate higher levels of problems and difficulties.

*Item fit* was determined based on a sample size adjusted cut-off [25]. With a total sample size of N = 201, Infit < 1.14 and Outfit < 1.42 support absence of underfit. The item fit statistics are shown in Table 1. Based on Outfit, all items of PARADISE 24 showed good fit. Two items showed Infit values above the acceptable cut-off, namely Item 3Problems with appetite (Infit = 1.33), and Item 15Walking a long distance (Infit = 1.24). The supplementary items from the EDEN 18 selection presented all good item fit. When analyzed jointly, Item 3Problems with Appetite showed the worst fit of all items (Infit = 1.41, Outfit = 1.44). A few other items presented Infit values above cut-off: Item 10Bodily pain (Infit = 1.19), Item 12Difficulty in remembering (Infit = 1.15), Item 15Walking a long distance (Infit = 1.23), and Item 17Sexual activities (Infit = 1.19).

**Figure 1:**
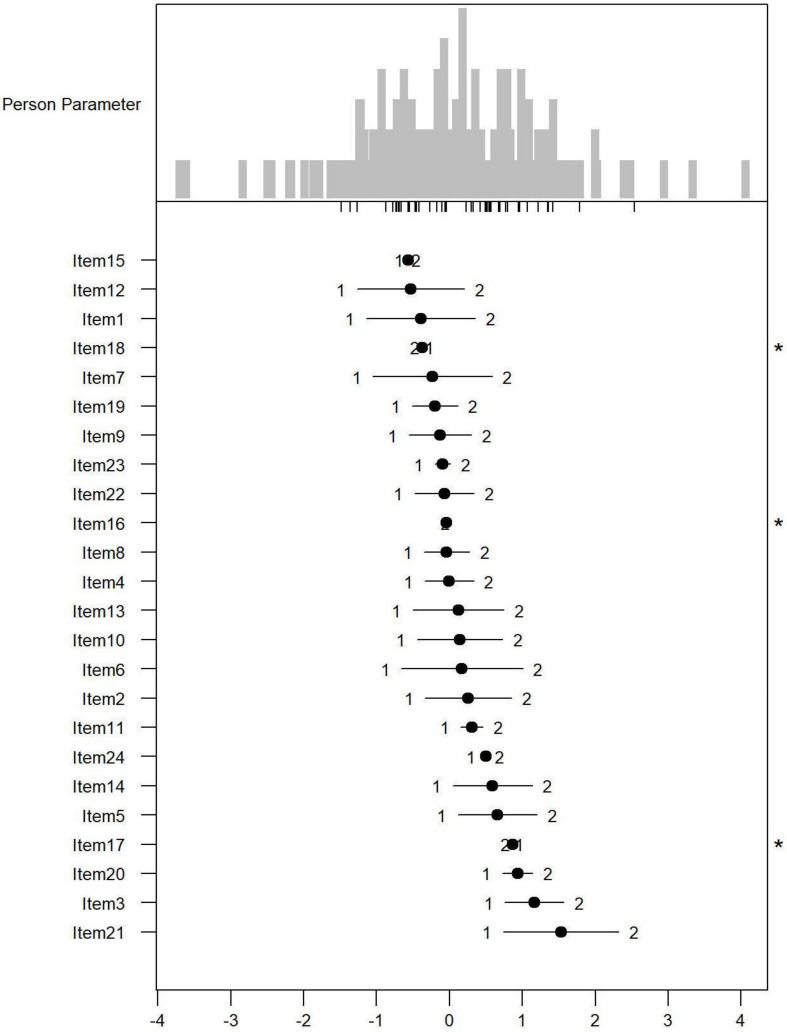
Person-item map for the person parameter and item parameter distribution of the 24 Paradise items having response categories (1 = none, 2 = mild to moderate, and 3 = severe to extreme PSDs). * Disordered thresholds are flagged by means of a ‘*’ in the margin of the figures.

**Figure 2:**
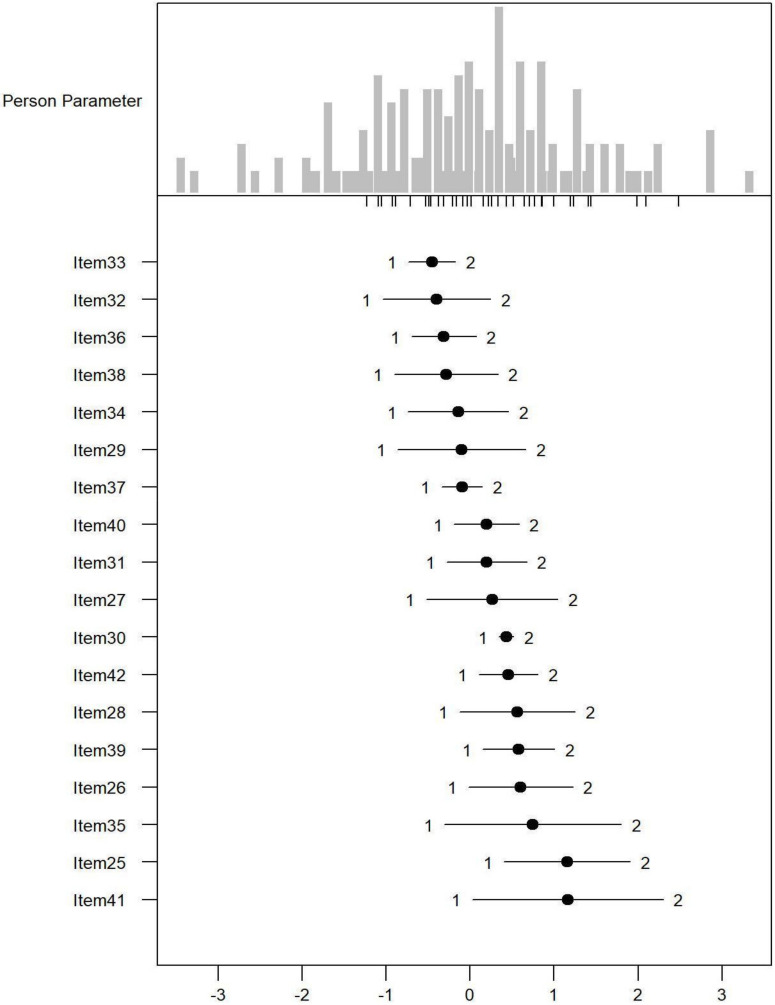
Person-item map for the person parameter and item parameter distribution of the 18 additional Eden items having three response categories (1 = none, 2 = mild to moderate, and 3 = severe to extreme PSDs).

**Figure 3:**
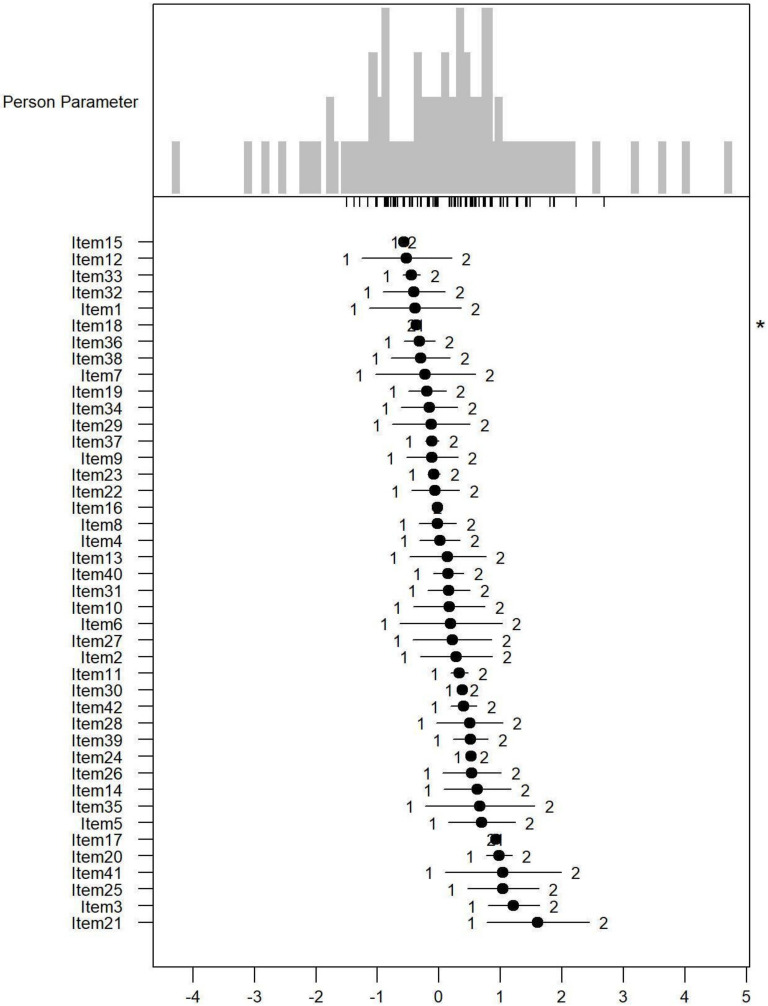
Person-item map for the person parameter and item parameter distribution of the Paradise and Eden items combined having three response categories (1 = none, 2 = mild to moderate, and 3 = severe to extreme PSDs). * Disordered thresholds are flagged by means of a ‘*’ in the margin of the figures.

*Monotonicity*: Figure 1, Figure 2 and Figure 3 show the person item maps of the distribution of the person parameter, i.e., the participants’ Rasch-based scores, and the distribution of the item parameter. Smaller item parameters indicate response categories of items that are more likely to be endorsed as being a problem. Smaller person parameters indicate persons with fewer PSDs. Three items of the PARADISE 24 scale presented disordered thresholds: Item 16 assessing ‘Problems in grooming, dressing, toileting or eating,’ Item 17 assessing ‘Difficulties in sexual activities, and Item 18 assessing ‘Difficulties in staying by yourself’ (Figure 1). All the items from the Eden-18 selection presented ordered difficulty thresholds (Figure 2). When analyzed jointly, only Item 17 ‘Difficulties in sexual activities’, and Item 18 ‘Difficulties in staying by yourself’ presented disordered thresholds again but the monotonicity of the remaining items did not suffer (Figure 3).

**Figure 4:**
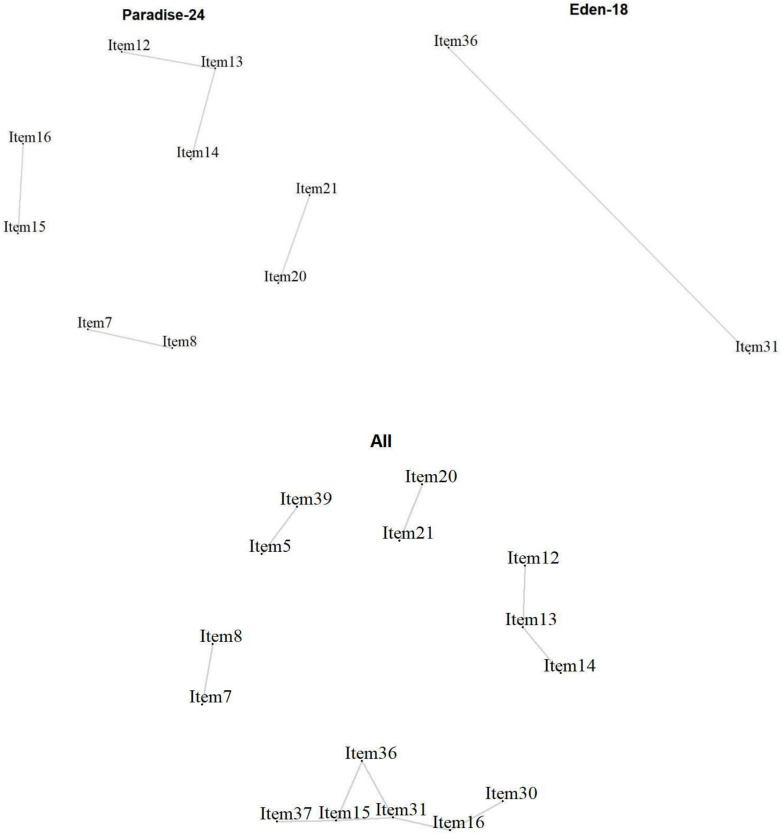
Local item dependencies above cut-off for the 24 Paradise items, the 18 additional Eden items, and the two combined.

*Local Item Dependencies:* All analyses showed locally dependent items (Figure 4). In the PARADISE 24 scale, Item 7 ‘Feeling sad, low or depressed’ and 8 ‘Worry or anxiety’ (*r* = 0.36) correlated above the cut-off. Item 20 ‘Initiating and maintaining a friendship’ and Item 21 ‘Getting along with people close to you’ (*r* = 0.49) also showed a high dependency, as well as Item 15 ‘Walking a long distance’, and Item 16 ‘Grooming, dressing, toileting, or eating’ (*r* = 0.32), Item 14 ‘Starting and maintaining a conversation’ and Item 13 ‘Making decisions’ (*r* = 0.32), as well as Item 13 with Item 12 ‘Remembering to do important things’ (*r* = 0.35). For the Eden-18 selection, only a couple of items showed a high dependency, namely Item 31 ‘Balance’ and Item 36 ‘Lifting and carrying things’ (r=0.31). When analyzed together, some dependencies are observed again, but with the occurrence of new dependencies across the two sets of items. Item 39 ‘Dealing with conflicts and tensions’ and Item 5 ‘Being so irritable that you started arguments and shouted or hit at people’ correlated highly (*r* = 0.36). The previously observed dependencies between Item 15 and Item 16 for PARADISE 24, and Item 31 and Item 36 for EDEN 18 were in one cluster with, in addition, Item 30 ‘Urinating and incontinence’ that correlated highly with Item 16 ‘Grooming, dressing, toileting, or eating’ (*r* = 0.37).

*Unidimensionality:* Unsurprisingly, at this stage the PARADISE 24 item set shows multidimensionality with the 1. eigenvalue of 2.81, explaining 11.7% of the variance. This is not unexpected, given the many item dependencies. A calibration using testlets may alleviate, probably even solve, this issue. The EDEN 18 item selection also indicates multidimensionality, with the 1. eigenvalue of 2.52 and a first component explaining 14.01% of the variance. Analysed jointly the dimensionality is high with a 1st component of 4 explaining 9.54% of the variance.

*Differential Item Functioning:* The DIF analysis tested subgroup effects of gender, age groups, data collection, diagnosis, and the living situation. No DIF was observed for age groups, gender, and diagnosis (Table 1). Paradise-24 Item 11 ‘Difficulty in concentrating for ten minutes’ showed DIF for the data collection, and Item 16 ‘Difficulty in grooming, dressing, toileting, or eating’ showed DIF for the data collection and living situation. Eden-18 Item 30 ‘Urinating and incontinence’ showed DIF for the data collection, Item 31 ‘Problem with balance,’ Item 32 ‘Difficulty in learning a new task,’ as well as Item 37 ‘Difficulty in moving around’ showed DIF for the living situation and data collection. The same and no additional items presented DIF in the joint analyses of the two sets of items.

## 4. Discussion

This study is a first attempt to create an ICF-based functioning metric for dementia. The psychometric analysis shows first that the PARADISE 24 and the selection of 18 additional condition-specific items can be expected to have good measurement properties to assess functioning in persons with dementia. A division of the items into subscales may be advised, given clusters of redundant items. On the other hand, the reduction of the length of the scale can be discussed in light of the dependencies. However, the number of dependent items is most significant in the PARADISE 24 metric in the assessment of persons with dementia.

The aim of the study was not only to investigate the metric properties of the PARADISE 24 items in a population with dementia but also to improve the scale by adding dementia-specific items (EDEN 18). Ultimately, the analysis should help reduce the EDEN 18 selection to twelve items based on the metric properties of the scale. However, based on this analysis, the choice of which item to discard from the supplementary 18-item selection is a challenge. The supplementary items selected for assessment in a population with dementia, the so-called Eden-18 items showed good measurement properties. The PCM analysis did not point to any item misfit, the response thresholds worked well, and reliability is good. In the co-calibration of the PARADISE-24 and EDEN-18, four items from the 18 items condition-specific selection showed strong dependencies: Item 30 ‘Urinating and incontinence,’ Item 31 ‘Balance’, Item 36 ‘Lifting Carrying’, and Item 37 ‘Moving around.’ With regard to Item 37 ‘Moving around,’ the PARADISE 24 scale has Item 15 ‘Difficulty in walking a long distance’ that also assesses mobility. If selecting between the two mobility items, it would be somewhat better to remove Item 15 from the PARADISE 24 selection, because it showed worse fit than Item 37. A selection may be also made between Item 31 ‘Balance’ and Item 36 ’Lifting and Carrying’, by only keeping Item 31 which is expected to have higher clinical relevance with persons with dementia. Understanding the difference in specific indices of balance and gait among patients with different degrees of cognitive impairments and healthy controls could help to develop better balance-oriented rehabilitation programs in older adults with early-stage cognitive impairment [36].

Other items could be good candidates for removal if the preservation of the PARADISE 24 scale is not the aim but rather a selection of items from the larger pool of PSD items. Some of the original items may not be as sound in a population with dementia as they presented a very high amount of missing values. These are Item 22 ‘Difficulty in day-to-day work or school,’ Item 17 ‘Difficulty with sexual activities’, with this item also presenting disordered thresholds. The prevalence of sexual dysfunction increases with age: cognitive impairment affects the frequency of and satisfaction with sexual activity, as well as the capacity to consent. The identification of sexual dysfunction in people with dementia in the early stage requires a clinical assessment of sexual behaviors and an understanding of the patient’s internal experience, which can be challenging and cannot be resolved with a single item. Research does suggest utility in the validation of specific assessment tools and habilitative, psychotherapeutic, and pharmacologic approaches specifically for this population [37]. This may explain the missing and disordered responses to Item 17 and the possibility of removing it, given the lack of reliability at this stage of the disease.

On the other hand, dementia-related inappropriate sexual behaviors are prevalent in the advanced stage of dementia and require management through both non-pharmacologic and pharmacologic approaches [38].

Item 18’Staying by yourself’ also had a noticeable number of missing values and showed disordered thresholds. The item with the worst fit was Item 3 ‘Appetite,’ which seems to be an item that is not frequently endorsed as being a big problem. It may be that this item does not make sense in this population as appetite is not directly correlated to dementia at these stages of the disease and only possibly makes sense at later stages of the disease when functioning is generally diminished. Prevalence of malnutrition and risk of malnutrition is higher in institutionalized elderly patients with advanced dementia and increases with the progression of the disease. A previous study confirmed that long-term care facility residents affected by severe cognitive impairment are characterized by a poor nutritional state, a serious impairment of functional conditions, and increased mortality [39]. An accurate evaluation of the nutritional status becomes necessary in the more advanced stages of the disease rather than in the early stages. Given that the new metric is targeted toward earlier stages of dementia, this item would be a good candidate for reducing the item pool.

Therefore, to fit a population with dementia, the statistical analysis indicated that the Item 3 ‘Appetite,’ Item 17 ‘Sexuality,’ Item 22 ‘Work or School,’ and Item 37 ‘Moving around’, or Item 15 ‘Walking 1 kilometer’ could be considered for removal. In any case, the clinical implications on the progression of disease suggest performing further studies in order to assess the possible contribution of appetite and sexuality for a better definition of a functional profile. Two further items from PARADISE 24 are also highly redundant, these are Item 20 ‘Initiating and maintaining friendship’ and Item 21 ‘Getting along with people close to you,’ and a selection between the two could be made. At this stage, we do not want to remove any items from the PARADISE 24 scale, however, as since the original scale was defined for a broader population with various brain disorders, the removal of items to fit a sample with only persons with dementia could be discussed and justified.

From a demographic point of view, this is a typical elderly population with a diagnosis of dementia. The most frequent diagnosis of dementia is vascular type according to the epidemiological data available for the elderly admitted to a nursing home, which is the prevalent condition in the population recruited in this research. A lower education level is considered a risk factor for the development of this disease, even if not all the studies agree with this topic [40]. It is also suggested that the relationship between education and dementia may not be unique to Alzheimer’s Disease, but the correlation between vascular dementia and education needs further ascertainment. However, we could refer to the correlation between stroke and education level that was found to be significant in previous research [41], considering that stroke and vascular dementia share risk factors that are quite the same.

Limitations: it is challenging to recruit a sample of patients with mild to moderate dementia, since a majority of these patients are still at home, cared for by family. Furthermore, this is a chronic condition, in which the patient turnover is extremely slow. The analysis of an assessment tool with up to 32 items based on a smaller sample of 200 persons with dementia could only be achieved by combining data from two different surveys that used the same assessment items. Fortunately, an analysis of differential item functioning did not show any effect of the combined survey.

It can be expected that the precision of parameter estimates at the extreme of the scales, where observations are less precise, is diminished. A validation of this analysis, in the future, with more data, will be necessary to further develop the metric.

## 5. Conclusions

The current study, as a first attempt to create an ICF-based functioning tool specific for dementia, has reported the findings of an exploratory psychometric analysis, showing that the PARADISE24 and the selection of 18 additional condition-specific items (EDEN18) have good measurement properties to assess functioning in persons with dementia. A proposal for a selection to reduce the number of additional items from 18 to 12 has been discussed. Future research with a larger sample will explore strategies to handle the item dependencies. A division of the item set into subscales may not be excluded. Future research, towards a smaller set of supplementary items, may also consider expert-based approaches to keep the most relevant items for this health condition. Given that some items showed different levels of difficulty according to the living situation, two versions of the tool for independent versus assisted living might also be considered.

Awareness of dementia as a public health problem has increased in more recent times, particularly in the last two decades. Even so, while it is quite well studied from a clinical point of view, there is a lack of understanding of functional ascertainment. The literature available is very limited and it is not sufficient to produce a theoretical framework. We hope with this research paper to stimulate a debate about the necessity of a deeper understanding of the functional issues associated with dementia.

## Figures and Tables

**Table 1:** Fit of items including infit and outfit statistic and presence of DIF for the 24 Paradise items, the 18 additional Eden items, and the two combined.

		PARADISE		EDEN		All	
Item	Question	In/Outfit	DIF	In/Outfit	DIF	In/Outfit	DIF
Item 1	Problem due to not feeling rested and refreshed during the day?	0.79/0.8				0.87/0.85	
Item 2	Problem with loss of interest?	0.86/0.89				0.96/0.98	
Item 3	Problem with your appetite?	1.23/1.33				1.44/1.41	
Item 4	Problem with sleeping, such as falling asleep, waking up frequently during the night or waking up too early in the morning?	1.14/1.02				1.35/1.09	
Item 5	Problem being so irritable that you started arguments, shouted at people or even hit people?	1.11/1.11				1.11/1.11	
Item 6	Problem with being slowed down or feeling as if things were moving too fast around you?	0.89/0.89				0.98/0.96	
Item 7	Problem with feeling sad, low or depressed?	0.88/0.91				0.96/0.99	
Item 8	Problem with worry or anxiety?	0.91/0.97				0.96/1.02	
Item 9	Problem with not being able to cope with all the things that you had to do?	0.77/0.8				0.83/0.88	
Item 10	How much bodily ache or pain did you have?	1.12/1.13				1.22/1.19	
Item 11	Difficulty in concentrating on doing something for ten minutes?	0.84/0.87	S			0.96/0.95	S
Item 12	Difficulty in remembering to do important things?	1.17/1.09				1.24/1.15	
Item 13	Difficulty in making decisions?	0.88/0.88				0.96/0.94	
Item 14	Difficulty in starting and maintaining a conversation?	1.07/1.07				1.11/1.11	
Item 15	Difficulty in walking a long distance such as a kilometer (or equivalent)?	1.28/1.25				1.24/1.23	
Item 16	Difficulty in grooming or dressing, toileting or eating?	0.94/0.98	R; S			0.85/0.9	R; S
Item 17	Difficulty in sexual activities?	1.1/1.06				1.21/1.19	
Item 18	Difficulty in staying by yourself for a few days?	0.79/0.84				0.88/0.9	
Item 19	Difficulty with looking after your health, such as eating well, exercising and taking your medicines?	0.87/0.88				0.94/0.92	
Item 20	Difficulty in initiating and maintaining a friendship?	1.04/0.97				1.12/1.02	
Item 21	Difficulty in getting along with people who are close to you?	0.85/0.86				0.89/0.89	
Item 22	Difficulty in your day-to-day work or school?	0.75/0.77				0.73/0.76	
Item 23	Difficulty with managing your money?	1.28/1.05				1.27/1.07	
Item 24	Difficulty in joining in community activities (for example, festivities, religious or other activities) in the same way as anyone else can?	1.12/1.08				1.19/1.1	
Item 25	Problem resisting doing or saying things in ways you would normally think were inappropriate?			1.1/1.08		0.97/0.98	
Item 26	Problem with feeling lonely even when with people?			0.93/0.98		0.91/0.97	
Item 27	Problem in finding the words you wanted to say or understanding words said to you?			0.96/1		0.91/0.96	
Item 28	Problem with feeling that your thoughts were too slow or that you could not think clearly?			0.98/0.87		0.92/0.88	
Item 29	Difficulty in analyzing and finding solutions to problems in day-to-day life?			0.8/0.83		0.78/0.82	
Item 30	Problem with passing water (urinating) or in controlling urine?			0.87/0.89	S	0.85/0.9	S
Item 31	Problem with your balance?			1.11/1.12	R; S	1.06/1.1	R; S
Item 32	Difficulty in learning a new task, for example, learning how to get to a new place?			1.05/1.05	R; S	0.99/1.03	R; S
Item 33	Difficulty in reading books, instructions or newspapers?			1.11/1.03		1.04/1.01	
Item 34	Difficulty in carrying out your day-to-day activities?			0.71/0.7		0.67/0.7	
Item 35	Difficulty in generally understanding what people say?			0.91/0.92		0.88/0.91	
Item 36	Difficulty in lifting and carrying things?			1.16/1.09		1.04/1.05	
Item 37	Difficulty with moving around?			1.21/1.13	R; S	1.15/1.11	R; S
Item 38	Difficulty in taking care of your household responsibilities?			0.87/0.85		0.86/0.85	
Item 39	Difficulty in dealing with conflicts and tensions with others?			0.96/0.97		0.88/0.93	
Item 40	Difficulty in providing for or supporting others?			0.92/0.94	R; S	0.92/0.9	R
Item 41	Difficulty in dealing with people you do not know?			1.16/1.14	R; S	1.08/1.07	R
Item 42	Difficulty in doing things by yourself for relaxation or pleasure?			0.88/0.85		0.83/0.82	

DIF: R = Residency (home versus inpatient care); S = Sample (Paradise or Eden).

**Table 2:** Sample Descriptives Stratified by Data Collection/Origin.

	Overall	PARADISE-Data	EDEN-Data	*p*-Value
N	201	80	121	
Age-mean (SD)	82.55 (6.95)	82.06 (5.51)	82.88 (7.78)	0.418
Gender: Female (%)	152 (76.0)	63 (78.8)	89 (74.2)	0.566
Marital Status				0.078
Married	53 (26.4)	25 (31.2)	28 (23.1)	
Widowed	114 (56.7)	48 (60.0)	66 (54.5)	
Divorced	12 (6.0)	2 (2.5)	10 (8.3)	
Never Married	22 (10.9)	5 (6.2)	17 (14.0)	
				<0.001
Education (%)				
Less than primary school		3 (3.8)		
Primary school completed		15 (18.8)		
Secondary school completed		3 (3.8)		
High school (or equivalent) completed		32 (40.0)		
University completed		22 (27.5)		
Post graduate degree completed		5 (6.2)		
Education (%)				
Illiteracy			33 (27.3)	
Primary School			67 (55.4)	
High School			16 (13.2)	
Degree			5 (4.1)	
Residency: Home (%)	103 (51.2)	70 (87.5)	33 (27.3)	<0.001
Diagnosis (%)				<0.001
Alzheimer Disease	86 (44.3)	49 (61.3)	37 (32.5)	
Vascular Dementia	92 (47.4)	27 (33.8)	65 (57.0)	
Other	16 (8.2)	4 (5.0)	12 (10.5)	
MMSE-mean (SD)	19.26 (3.79)	21.10 (2.89)	18.03 (3.83)	<0.001
Satisfaction in ADL (%)				<0.001
Very satisfied		3 (3.8)		
Satisfied		27 (33.8)		
Neither satisfied nor dissatisfied		33 (41.2)		
Dissatisfied		17 (21.2)		
Independence in ADL (%)				
Independence			14 (11.7)	
Mild dependence			23 (19.2)	
Moderate dependence			33 (27.5)	
Severe dependence			50 (41.7)	

**Table 3:** Model fit including person-item targeting and reliability indices for the 24 Paradise items, the 18 additional Eden items, and the two combined.

			Mean	SD	(Min; Max)	Mean Residuals	SD Residuals
Paradise Items	Targeting	Difficulty	0.19	0.86	(−1.48; 2.54)	0.2	0.04
		Ability	0.11	0.97	(−3.71; 4.06)	0.33	0.08
			Index	Coefficient	Strata		
	Reliability	Difficulty	0.94	4.17	5.89		
		Ability	0.9	3.07	4.43		
			Alpha				
		Test	0.91				
			**Mean**	**SD**	**(Min; Max)**	**Mean Residuals**	**SD Residuals**
Eden Items	Targeting	Difficulty	0.25	0.95	(−1.23; 2.49)	0.2	0.03
		Ability	0.02	1.21	(−4.25; 4.3)	0.41	0.11
			Index	Coefficient	Strata		
	Reliability	Difficulty	0.88	4.69	6.58		
		Ability	0.96	2.8	4.07		
			Alpha				
		Test	0.91				
			**Mean**	**SD**	**(Min; Max)**	**Mean Residuals**	**SD Residuals**
All Items	Targeting	Difficulty	0.21	0.88	(−1.5; 2.69)	0.2	0.03
		Ability	0.16	0.98	(−4.28; 4.7)	0.25	0.09
			Index	Coefficient	Strata		
	Reliability	Difficulty	0.95	4.29	6.05		
		Ability	0.94	4.31	6.08		
			Alpha				
		Test	0.95				

## Data Availability

Data are available on a digital devise belonging to ANASTE Humanitas Foundation, in Via dei Gracchi 137, Rome (Italy).

## Appendix A

**Table A1:** Response frequencies (percentages) for all the selected items, the 24 Paradise items, and the 18 additional Eden items with significance test on the response frequencies per item (*p*-value).

Item	Option	All	PARADISE 24 Data	EDEN18 Data	*p*-Value
Item 1	None	40 (19.9)	13 (16.2)	27 (22.3)	<0.001
	Mild to moderate	93 (46.3)	55 (68.8)	38 (31.4)	
	Severe to extreme	68 (33.8)	12 (15.0)	56 (46.3)	
Item 2	None	68 (33.8)	25 (31.2)	43 (35.5)	0.001
	Mild to moderate	85 (42.3)	46 (57.5)	39 (32.2)	
	Severe to extreme	46 (22.9)	8 (10.0)	38 (31.4)	
	Missing	2 (1.0)	1 (1.2)	1 (0.8)	
Item 3	None	115 (57.2)	48 (60.0)	67 (55.4)	0.043
	Mild to moderate	62 (30.8)	28 (35.0)	34 (28.1)	
	Severe to extreme	24 (11.9)	4 (5.0)	20 (16.5)	
Item 4	None	64 (31.8)	34 (42.5)	30 (24.8)	0.004
	Mild to moderate	75 (37.3)	31 (38.8)	44 (36.4)	
	Severe to extreme	62 (30.8)	15 (18.8)	47 (38.8)	
Item 5	None	88 (43.8)	37 (46.2)	51 (42.1)	0.074
	Mild to moderate	78 (38.8)	35 (43.8)	43 (35.5)	
	Severe to extreme	35 (17.4)	8 (10.0)	27 (22.3)	
Item 6	None	58 (28.9)	21 (26.2)	37 (30.6)	0.026
	Mild to moderate	97 (48.3)	47 (58.8)	50 (41.3)	
	Severe to extreme	45 (22.4)	11 (13.8)	34 (28.1)	
	Missing	1 (0.5)	1 (1.2)	0 (0.0)	
Item 7	None	44 (21.9)	19 (23.8)	25 (20.7)	0.001
	Mild to moderate	97 (48.3)	50 (62.5)	47 (38.8)	
	Severe to extreme	59 (29.4)	11 (13.8)	48 (39.7)	
	Missing	1 (0.5)	0 (0.0)	1 (0.8)	
Item 8	None	63 (31.3)	28 (35.0)	35 (28.9)	<0.001
	Mild to moderate	74 (36.8)	43 (53.8)	31 (25.6)	
	Severe to extreme	64 (31.8)	9 (11.2)	55 (45.5)	
Item 9	None	56 (27.9)	26 (32.5)	30 (24.8)	0.017
	Mild to moderate	79 (39.3)	38 (47.5)	41 (33.9)	
	Severe to extreme	65 (32.3)	16 (20.0)	49 (40.5)	
	Missing	1 (0.5)	0 (0.0)	1 (0.8)	
Item 10	None	64 (31.8)	25 (31.2)	39 (32.2)	0.064
	Mild to moderate	86 (42.8)	41 (51.2)	45 (37.2)	
	Severe to extreme	51 (25.4)	14 (17.5)	37 (30.6)	
Item 11	None	82 (40.8)	46 (57.5)	36 (29.8)	<0.001
	Mild to moderate	65 (32.3)	28 (35.0)	37 (30.6)	
	Severe to extreme	53 (26.4)	5 (6.2)	48 (39.7)	
	Missing	1 (0.5)	1 (1.2)	0 (0.0)	
Item 12	None	36 (17.9)	7 (8.8)	29 (24.0)	<0.001
	Mild to moderate	91 (45.3)	49 (61.3)	42 (34.7)	
	Severe to extreme	74 (36.8)	24 (30.0)	50 (41.3)	
Item 13	None	60 (29.9)	21 (26.2)	39 (32.2)	0.005
	Mild to moderate	86 (42.8)	40 (50.0)	46 (38.0)	
	Severe to extreme	50 (24.9)	14 (17.5)	36 (29.8)	
	Missing	5 (2.5)	5 (6.2)	0 (0.0)	
Item 14	None	84 (41.8)	25 (31.2)	59 (48.8)	<0.001
	Mild to moderate	79 (39.3)	46 (57.5)	33 (27.3)	
	Severe to extreme	37 (18.4)	8 (10.0)	29 (24.0)	
	Missing	1 (0.5)	1 (1.2)	0 (0.0)	
Item 15	None	46 (22.9)	27 (33.8)	19 (15.7)	<0.001
	Mild to moderate	53 (26.4)	30 (37.5)	23 (19.0)	
	Severe to extreme	94 (46.8)	22 (27.5)	72 (59.5)	
	Missing	8 (4.0)	1 (1.2)	7 (5.8)	
Item 16	None	74 (36.8)	45 (56.2)	29 (24.0)	<0.001
	Mild to moderate	52 (25.9)	24 (30.0)	28 (23.1)	
	Severe to extreme	75 (37.3)	11 (13.8)	64 (52.9)	
Item 17	None	63 (31.3)	6 (7.5)	57 (47.1)	<0.001
	Mild to moderate	24 (11.9)	5 (6.2)	19 (15.7)	
	Severe to extreme	21 (10.4)	1 (1.2)	20 (16.5)	
	Missing	93 (46.3)	68 (85.0)	25 (20.7)	
Item 18	None	48 (23.9)	17 (21.2)	31 (25.6)	<0.001
	Mild to moderate	42 (20.9)	26 (32.5)	16 (13.2)	
	Severe to extreme	79 (39.3)	19 (23.8)	60 (49.6)	
	Missing	32 (15.9)	18 (22.5)	14 (11.6)	
Item 19	None	56 (27.9)	23 (28.7)	33 (27.3)	0.010
	Mild to moderate	74 (36.8)	38 (47.5)	36 (29.8)	
	Severe to extreme	71 (35.3)	19 (23.8)	52 (43.0)	
Item 20	None	107 (53.2)	38 (47.5)	69 (57.0)	0.006
	Mild to moderate	58 (28.9)	31 (38.8)	27 (22.3)	
	Severe to extreme	31 (15.4)	7 (8.8)	24 (19.8)	
	Missing	5 (2.5)	4 (5.0)	1 (0.8)	
Item 21	None	118 (58.7)	41 (51.2)	77 (63.6)	<0.001
	Mild to moderate	68 (33.8)	39 (48.8)	29 (24.0)	
	Severe to extreme	15 (7.5)	0 (0.0)	15 (12.4)	
Item 22	None	29 (14.4)	6 (7.5)	23 (19.0)	<0.001
	Mild to moderate	49 (24.4)	20 (25.0)	29 (24.0)	
	Severe to extreme	46 (22.9)	0 (0.0)	46 (38.0)	
	Missing	77 (38.3)	54 (67.5)	23 (19.0)	
Item 23	None	62 (30.8)	29 (36.2)	33 (27.3)	<0.001
	Mild to moderate	61 (30.3)	31 (38.8)	30 (24.8)	
	Severe to extreme	60 (29.9)	9 (11.2)	51 (42.1)	
	Missing	18 (9.0)	11 (13.8)	7 (5.8)	
Item 24	None	94 (46.8)	37 (46.2)	57 (47.1)	0.474
	Mild to moderate	55 (27.4)	26 (32.5)	29 (24.0)	
	Severe to extreme	47 (23.4)	15 (18.8)	32 (26.4)	
	Missing	5 (2.5)	2 (2.5)	3 (2.5)	
Item 25	None	102 (50.7)	50 (62.5)	52 (43.0)	0.004
	Mild to moderate	72 (35.8)	27 (33.8)	45 (37.2)	
	Severe to extreme	26 (12.9)	3 (3.8)	23 (19.0)	
	Missing	1 (0.5)	0 (0.0)	1 (0.8)	
Item 26	None	83 (41.3)	41 (51.2)	42 (34.7)	0.007
	Mild to moderate	77 (38.3)	32 (40.0)	45 (37.2)	
	Severe to extreme	40 (19.9)	7 (8.8)	33 (27.3)	
	Missing	1 (0.5)	0 (0.0)	1 (0.8)	
Item 27	None	64 (31.8)	26 (32.5)	38 (31.4)	0.008
	Mild to moderate	88 (43.8)	43 (53.8)	45 (37.2)	
	Severe to extreme	48 (23.9)	10 (12.5)	38 (31.4)	
	Missing	1 (0.5)	1 (1.2)	0 (0.0)	
Item 28	None	79 (39.3)	39 (48.8)	40 (33.1)	0.001
	Mild to moderate	80 (39.8)	33 (41.2)	47 (38.8)	
	Severe to extreme	40 (19.9)	6 (7.5)	34 (28.1)	
	Missing	2 (1.0)	2 (2.5)	0 (0.0)	
Item 29	None	49 (24.4)	18 (22.5)	31 (25.6)	0.096
	Mild to moderate	86 (42.8)	39 (48.8)	47 (38.8)	
	Severe to extreme	59 (29.4)	18 (22.5)	41 (33.9)	
	Missing	7 (3.5)	5 (6.2)	2 (1.7)	
Item 30	None	88 (43.8)	51 (63.7)	37 (30.6)	<0.001
	Mild to moderate	57 (28.4)	26 (32.5)	31 (25.6)	
	Severe to extreme	54 (26.9)	3 (3.8)	51 (42.1)	
	Missing	2 (1.0)	0 (0.0)	2 (1.7)	
Item 31	None	70 (34.8)	37 (46.2)	33 (27.3)	<0.001
	Mild to moderate	75 (37.3)	40 (50.0)	35 (28.9)	
	Severe to extreme	54 (26.9)	3 (3.8)	51 (42.1)	
	Missing	2 (1.0)	0 (0.0)	2 (1.7)	
Item 32	None	42 (20.9)	11 (13.8)	31 (25.6)	0.008
	Mild to moderate	80 (39.8)	36 (45.0)	44 (36.4)	
	Severe to extreme	74 (36.8)	28 (35.0)	46 (38.0)	
	Missing	5 (2.5)	5 (6.2)	0 (0.0)	
Item 33	None	48 (23.9)	27 (33.8)	21 (17.4)	<0.001
	Mild to moderate	64 (31.8)	35 (43.8)	29 (24.0)	
	Severe to extreme	81 (40.3)	18 (22.5)	63 (52.1)	
	Missing	8 (4.0)	0 (0.0)	8 (6.6)	
Item 34	None	54 (26.9)	22 (27.5)	32 (26.4)	0.001
	Mild to moderate	81 (40.3)	43 (53.8)	38 (31.4)	
	Severe to extreme	66 (32.8)	15 (18.8)	51 (42.1)	
Item 35	None	76 (37.8)	31 (38.8)	45 (37.2)	0.033
	Mild to moderate	94 (46.8)	43 (53.8)	51 (42.1)	
	Severe to extreme	31 (15.4)	6 (7.5)	25 (20.7)	
Item 36	None	52 (25.9)	20 (25.0)	32 (26.4)	0.012
	Mild to moderate	71 (35.3)	38 (47.5)	33 (27.3)	
	Severe to extreme	75 (37.3)	22 (27.5)	53 (43.8)	
	Missing	3 (1.5)	0 (0.0)	3 (2.5)	
Item 37	None	62 (30.8)	43 (53.8)	19 (15.7)	<0.001
	Mild to moderate	65 (32.3)	32 (40.0)	33 (27.3)	
	Severe to extreme	70 (34.8)	5 (6.2)	65 (53.7)	
	Missing	4 (2.0)	0 (0.0)	4 (3.3)	
Item 38	None	47 (23.4)	16 (20.0)	31 (25.6)	<0.001
	Mild to moderate	80 (39.8)	49 (61.3)	31 (25.6)	
	Severe to extreme	66 (32.8)	13 (16.2)	53 (43.8)	
	Missing	8 (4.0)	2 (2.5)	6 (5.0)	
Item 39	None	87 (43.3)	39 (48.8)	48 (39.7)	0.007
	Mild to moderate	69 (34.3)	32 (40.0)	37 (30.6)	
	Severe to extreme	44 (21.9)	8 (10.0)	36 (29.8)	
	Missing	1 (0.5)	1 (1.2)	0 (0.0)	
Item 40	None	64 (31.8)	19 (23.8)	45 (37.2)	<0.001
	Mild to moderate	66 (32.8)	27 (33.8)	39 (32.2)	
	Severe to extreme	56 (27.9)	19 (23.8)	37 (30.6)	
	Missing	15 (7.5)	15 (18.8)	0 (0.0)	
Item 41	None	89 (44.3)	31 (38.8)	58 (47.9)	0.041
	Mild to moderate	87 (43.3)	40 (50.0)	47 (38.8)	
	Severe to extreme	22 (10.9)	6 (7.5)	16 (13.2)	
	Missing	3 (1.5)	3 (3.8)	0 (0.0)	
Item 42	None	83 (41.3)	35 (43.8)	48 (39.7)	<0.001
	Mild to moderate	67 (33.3)	37 (46.2)	30 (24.8)	
	Severe to extreme	49 (24.4)	6 (7.5)	43 (35.5)	
	Missing	2 (1.0)	2 (2.5)	0 (0.0)	
